# Artificial Intelligence in Nuclear Cardiology

**DOI:** 10.3390/jcm14186416

**Published:** 2025-09-11

**Authors:** Roberto Sciagrà, Samuele Valente, Marco Dominietto

**Affiliations:** 1 Nuclear Medicine, Department of Experimental and Clinical Biomedical Sciences, University of Florence, Careggi University Hospital, 50134 Florence, Italy; samuele.valente@unifi.it; 2 Gate To Brain SA, 6830 Chiasso, Switzerland; marco.dominietto@gatetobrain.com

**Keywords:** artificial intelligence, deep learning, machine learning, myocardial perfusion imaging

## Abstract

**Background/Objectives**: Artificial Intelligence (AI) is becoming increasingly important in Medicine. The aim of this review is to summarize its use in the field of Nuclear Cardiology. **Methods**: First, we provide a short description of how AI works. Then we performed a review of the literature focusing on the articles in which AI is used for image interpretation for diagnostic or prognostic purposes. **Results**: AI has been applied according to various approaches for both diagnosis and prognosis. The achieved gains have been so far relatively limited as compared to traditional methodologies. However, promising results have been reported, including interesting perspectives for the explainability of AI results and their potential integration in clinical routine. **Conclusions**: AI is soon going to play an important role in Nuclear Cardiology, but further improvements are needed to reach significant gains in terms of diagnostic accuracy, and prospective studies on its prognostic capabilities are still lacking. Furthermore, several important issues must be solved, such as availability and feasibility within the processing workflow, explainability, liability, and ethics of its application in clinical decision-making.

## 1. Introduction: Artificial Intelligence in Medicine

Artificial Intelligence (AI) is increasingly transforming the landscape of medicine by providing advanced tools to assist clinicians in diagnosing diseases, stratifying patients by disease severity, and predicting clinical outcomes with unprecedented speed and accuracy [1,2]. At its core, AI refers to computer systems that can perform tasks typically requiring human intelligence, such as recognizing patterns, learning from experience, and making informed decisions.

AI learns by analyzing vast amounts of data. In medicine, this typically involves medical images, electronic health records, genetic information, and clinical readouts. By processing these data, AI systems identify complex patterns and relationships that may be imperceptible to human observers. This learning process is achieved through algorithms that improve their performance over time as they are exposed to more examples—a process known as machine learning (ML) [3].

ML is a subset of AI that focuses on training algorithms to make predictions or decisions without being explicitly programmed for each specific task. ML encompasses a variety of algorithms, including decision trees, support vector machines, random forests, and ensemble techniques such as boosting and bagging [4,5]. Traditional ML models often require structured data and rely on manually engineered features to achieve optimal performance [4]. In contrast, deep learning (DL), a specialized subfield of ML, utilizes layered artificial neural networks capable of learning hierarchical feature representations from data [5,6]. Convolutional neural networks (CNNs), recurrent networks, long short-term memory units, and transformer-based models are increasingly used in medical imaging, speech analysis, and longitudinal data interpretation [7]. Training these models typically involves backpropagation, gradient descent optimization, and the use of activation and cost functions to reduce prediction error [7]. Modern architectures often integrate components such as attention mechanisms, skip connections, or transfer learning strategies to boost performance and adaptability with limited labeled data [7].

Other methods include unsupervised learning models, such as k-means clustering or autoencoders, which are used for identifying patient subgroups or latent patterns in medical imaging and clinical datasets without labeled data [8,9]. These tools vary in complexity, performance, and interpretability, and their suitability often depends on the type of data and the specific clinical question being addressed.

The learning process typically involves dividing the available dataset into three distinct subsets: the training set, the validation set, and the test set [6]. The training set is used to teach the AI model by allowing it to recognize patterns and learn relationships within the data. The validation set helps fine-tune the model by evaluating its performance during training and adjusting parameters to prevent overfitting [7]. Finally, the test set is used to independently assess the model’s generalization ability on previously unseen data. The performance of an AI model is often evaluated using various metrics, with the confusion matrix being a fundamental tool [10]. The confusion matrix provides a detailed breakdown of the model’s predictions, including true positives, true negatives, false positives, and false negatives. This allows researchers to calculate important performance indicators such as accuracy, sensitivity, specificity, and precision—metrics that are critical in medical applications where diagnostic errors can have significant consequences [9].

The applications of AI in medicine are diverse and expanding rapidly. AI-powered algorithms are now employed to detect diseases such as cancer, cardiovascular conditions, and neurological disorders from imaging modalities like magnetic resonance imaging (MRI), computed tomography (CT), and X-rays [11,12]. They assist in classifying disease severity, which supports treatment planning and risk stratification [13]. Furthermore, AI models can predict patient outcomes by analyzing historical and real-time data, contributing to personalized medicine and improving prognostic accuracy [14].

Beyond nuclear cardiology, AI is demonstrating remarkable impact across other domains of cardiovascular medicine, particularly in imaging and electrocardiography (ECG) analysis. In echocardiography, CNNs have been applied for automated view classification, quantification of cardiac function, and disease detection, including heart failure with preserved ejection fraction [15]. Cardiac MRI and CT have also benefited from AI-powered segmentation, plaque characterization, and risk stratification models [16]. In the realm of ECG analysis, deep learning algorithms have achieved expert-level performance in arrhythmia classification, atrial fibrillation detection, and even prediction of asymptomatic left ventricular dysfunction directly from 12-lead ECG data [17,18]. These systems enable scalable, real-time analysis of high-throughput data, facilitating earlier diagnosis, triage in emergency settings, and remote monitoring [19]. Together, these advances underscore the versatility and transformative potential of AI across all major cardiovascular diagnostic platforms.

## 2. AI in Nuclear Cardiology

### 2.1. General Considerations

**Figure 1:**
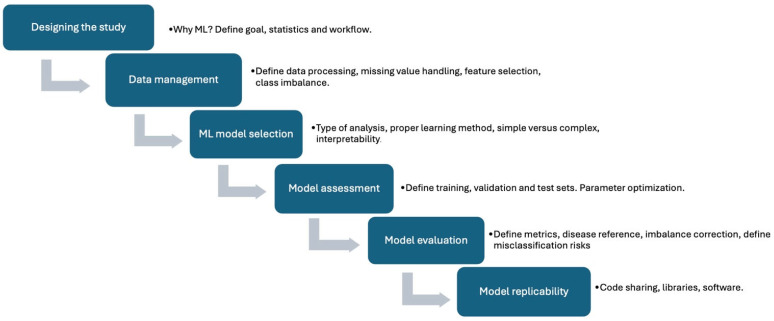
Schematic representation of the main points in the PRIME checklist, based on reference [20].

The applications of AI in medicine are manifolds and could represent major progress. However, since they concern human health, they should be thoroughly validated, fulfilling adequate methodological requirements. Accordingly, scientific societies and expert groups have already proposed proper guidelines. A most important one is the Proposed Requirements for Cardiovascular Imaging-Related Machine Learning Evaluation (PRIME) [20]. PRIME identifies the necessary steps to correctly perform studies on AI applications in the field of cardiovascular imaging. These are as well the criteria to assess the methodological reliability of published studies. More recently, in a position paper on AI in multimodality cardiovascular imaging endorsed by the European Association of Nuclear Medicine (EANM) and the European Association of Cardiovascular Imaging (EACVI), the key elements for an effective employment of AI in nuclear cardiology have been presented [21]. Figure 1 summarizes some important points to be considered.

AI-based techniques have been extensively implemented in scintigraphic image acquisition and processing. The focus of this review, however, will be on AI applications for the interpretation of nuclear cardiology images and for their integration with other patient data for diagnostic and prognostic purposes. For this aim, we examined the published literature on AI and nuclear cardiology and on AI and myocardial perfusion imaging (MPI) and retrieved articles from the reference lists of relevant publications; finally, we selected the papers on diagnostic and prognostic uses of AI-based methods.

### 2.2. Historical Perspective

Although AI is considered a quite recent development in the field of medicine in general, and of diagnostic imaging in particular, its use in the setting of nuclear cardiology techniques dates to many years ago. It was already in 1992 when Fujita and coworkers employed a neural network with the aim of helping the physician to detect abnormalities in ^201^Tl myocardial perfusion single photon emission computed tomography (SPECT) polar maps. The neural network was trained with 58 polar maps coupled to their correct interpretation, and then its classification in 16 further cases was compared with that proposed by unexperienced and experienced readers. The results showed a slight superiority over the unexperienced observer but remained inferior to those achieved by the experienced reader. As expected, increasing the complexity of the neural network and the number of training iterations improved the accuracy, but computing capabilities were then still a limitation in terms of processing time [22]. The very modern approach of that paper was not followed by other similar experiences in the subsequent years.

At the beginning of this century, automation in image interpretation was performed by case-based reasoning. This method implies the construction of a case library of images interpreted by experienced readers; then, the individual study is compared with the library, and the diagnostic interpretation is based on that of the five most similar cases. In the article by Khorsand and coworkers, the case-based reading was compared with the Cedar Sinai polar map analysis and with the visual interpretation by an experienced observer. The results of the three approaches were similar, and no significant diagnostic gain was achieved [23].

Another approach dating back to the end of the last century was the knowledge-based expert system, in which a series of interpretative rules derived from the observer analysis of images with known diagnoses was used to classify the results of myocardial perfusion scans. In their comparative study, Garcia and coworkers could demonstrate that the expert system obtained a reasonable accuracy when the reference was the interpretation by an experienced nuclear physician, whilst the agreement was lower using coronary angiography as the gold standard. However, the human expert, too, was less effective when compared with coronary angiography [24].

A further development of this approach by the same research group has been to construct an AI-driven structured report. As in previous studies, the reference standard was the consensus of experts. The AI-driven structured report was not inferior to the consensus expert reading only if the setting of high specificity was chosen. Otherwise, both in the case of trade-off of sensitivity/specificity and of high-sensitivity settings, there were significant differences between AI-driven reports and the reference standard, which were also clearly higher than those between an additional independent expert and the consensus reference [25].

### 2.3. ML and DL: Diagnosis of CAD

The overall disappointing results of the above-mentioned approaches together with the overflowing AI evolution opened the way to the extensive use of ML- and DL-based models for improving the interpretation of nuclear cardiology examinations. However, the first applications of ML to MPI in the diagnostic setting did not reach more effective results than those reported by case-based or knowledge-based methodologies. Arsanjani and coworkers enrolled more than one thousand patients and submitted their MPIs and clinical data to a Logit-Boost ML algorithm to identify coronary artery disease (CAD) [26]. The achieved diagnostic accuracy was equal to that of one of the experts and just slightly superior to another observer, and to the established total perfusion defect (TPD) approach. In general, the differences among ML and the other classifications were less than 5% [26]. In another study, a direct comparison between DL-based and knowledge-based approaches was performed in the detection of MPI abnormalities, using as reference the interpretation by two experienced readers. The two approaches reached high levels of sensitivity, specificity, and accuracy, but it is notable that among various pretrained networks used for DL, there were remarkable differences [27].

Indeed, even more-advanced AI applications such as ML and DL have potential pitfalls, as clearly indicated in the above-mentioned guidelines and position papers [20,21].

Problems can be related to the definition of the reference standard. It is interesting to compare two studies, which apparently performed a very similar analysis, respectively, on 566 and 1007 polar maps. Although they used almost the same algorithms, the best accuracy achieved by ML was quite different, 76.5% versus 93%. One of the explanations is that in the former study the reference standard was coronary angiography, and several clinical features were included in the model, whilst in the latter it was the consensus of two experienced readers, and only MPI parameters were considered [28,29]. Thus, the same AI approach can obtain quite different results if applied to different contexts, and this makes it accordingly difficult to define the true reliability of these methodologies. Another problem can arise when there is a selection bias in the training population, for instance because of the prevalence of high-risk patients, which in turn can produce a disease overestimation in the testing cohort. To overcome this bias, data-augmentation techniques have been proposed, with the aim of rebalancing the case distribution in the training set to make it closer to the testing population. Miller and coworkers could thus achieve an improvement in the AI model classification over that obtained using the original population. However, it is quite disappointing to remark that the best AI classification after selection bias correction was just slightly better than that reached by the traditional TPD approach [30]. In very large patient populations in which several variables are included in the AI model, problems arise when there are missing data. There are various possible approaches to handle this problem, but probably the most effective is the removal of the involved variables [31].

In the last years, several papers have been published presenting ML- or DL-based models for the purpose of improving the diagnosis of CAD (Table 1), but a large majority of them are based on relatively small cohorts, making it difficult to achieve an adequate algorithm generalizability, because in too small patient cohorts the training results may be eventually very accurate in the dataset itself but useless in another population due to overfitting [32].

Nevertheless, some of these under-dimensioned studies deserve to be mentioned because they deal with interesting aspects of the potential use of AI in nuclear cardiology. For instance, there were studies specifically constructed to evaluate the capability of AI-based models to support the inexperienced observer in the assessment of MPI images. According to Chiba and coworkers, ML permitted the beginners to achieve the same level of diagnostic accuracy as experts, particularly in the classification of cases with relatively limited perfusion abnormalities [33]. In a more recent study, Kiso et al. used ML to predict the summed stress score (SSS) and the summed difference score (SDS) and compared the results with experienced observers. The main finding was a close correlation between the scores, which was more remarkable for the intermediate ones, where there is greater uncertainty [34]. The concept of using AI to improve the calculation of perfusion scores, such as TPD and SSS, has been applied by others with interesting results and the major advantage of a more straightforward implementation in the current processing pipeline [35]. Miller and coworkers examined whether an explainable DL model was able to improve the observer capability for CAD detection. They demonstrated that the diagnostic accuracy of the observer plus DL (area under the curve—AUC 0.779) was significantly higher than that of the observer alone (0.747) and of stress TPD (0.718) [36]. The most interesting point of this study is that it could represent a reasonable implementation of AI in the diagnostic setting, since providing a single patient with DL-based probability maps would increase the observer’s reliability without limiting the final clinical judgement. A particular study in which the focus has been on functional parameters is that by Zhang, who used DL to improve the visual assessment of wall motion from MPI images to be compared with that of human observers having as reference the echocardiographic evaluation by experienced cardiologists of the same patients [37,38]. There was a significant gain in accuracy using DL (AUC 0.82) versus the human reader (AUC 0.77, *p* < 0.001). Moreover, DL was able to measure left ventricular ejection fraction more accurately than the standard software.

Because of the cohort dimension problem, a central role for the development of AI applications in nuclear cardiology has been played by the creation of a large registry of MPI scans acquired with cadmium-zinc-telluride (CZT) SPECT and REFINE (Registry of Fast Myocardial Perfusion Imaging with Next generation SPECT) [38]. Using the data collected by REFINE, several papers have explored the capabilities of different AI approaches for diagnosis and prognosis. Most recently, REFINE has been updated with REFINE 2.0 [39].

The first goal of the REFINE-based studies has been to improve the diagnosis of CAD using DL, as shown by the article by Betancur and coworkers [40]. Similarly, the same group applied AI to analyze the combination of upright and supine images acquired by CZT cameras. The results confirmed the superiority of this approach over the established TPD method [41].

A particular setting in which the use of AI could be advantageous for supporting the physician’s choice is the interpretation of stress-only MPI. Liu and coworkers applied a deep convolution neural network to a very large population (37,243 patients) and demonstrated higher accuracy and specificity than the traditional defect size approach, although the sensitivity was slightly lower [42]. On the other hand, using ML, Miller and coworkers tried to predict the pre-test probability of an abnormal MPI result in patients of the REFINE registry, considering 30 clinical features. The model was more effective than the standard ones, such as Diamond–Forrester [43].

**Figure 2:**
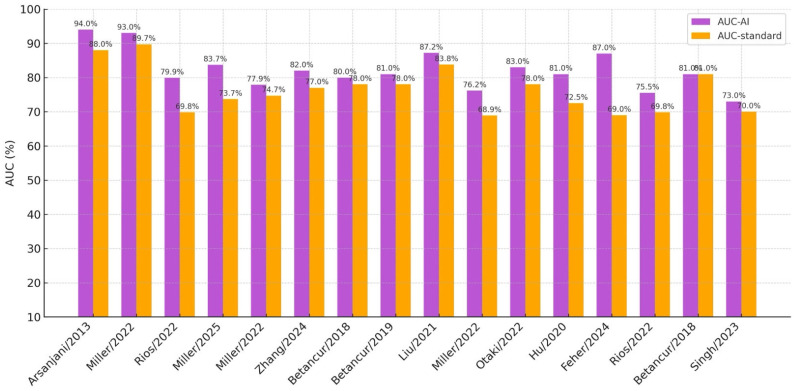
Histogram of the result metrics comparing AI AUC with the AUC of the reference methods.

Getting a significant superiority over traditional methods is the ultimate target of AI techniques, but another important goal to be reached is the implementation of explainability methods, which, as already mentioned, make it possible for the physician and for the patient to understand how AI has classified the single study, which consequently could improve the acceptance of AI-derived clinical decisions. In their study using DL on a quite large set of patients, Otaki and coworkers provided both an attention polar map that highlighted the region deemed abnormal by DL and a CAD probability map conveyed in the established 17-segment American Heart Association (AHA) scheme. They reported that DL reached a significantly larger AUC than the traditional TPD or the experienced reader, with the further advantages of an easy implementation in standard clinical software and of a short calculation time [44]. Despite the large patient cohorts made possible by REFINE, the final gain of AI models applied for diagnostic purposes over traditional methods has been always quite limited (Figure 2).

### 2.4. ML and DL: Prognosis of CAD

A field in which the use of AI could be more effective is prognostication, since AI allows the combination of multiple parameters beyond the MPI polar maps (Table 1). REFINE has been the basis of various studies about prognosis. Hu and coworkers have explored the capability of ML to predict the occurrence of early revascularization in CAD patients. The ML used 18 clinical, 9 stress, and 28 imaging variables and outperformed TPD and nuclear cardiologist expert interpretation. Moreover, even in this setting, the Authors were able to provide a method for making explainable the ML classification [45]. In another paper, Feher et al. focused on the prediction of heart failure, demonstrating that a DL model including clinical risk factors, stress variables, SPECT imaging, and calcium score (generated as well by DL on attenuation CT scans) was significantly superior to left ventricular ejection fraction and to a model based just on clinical variables [46]. A most interesting feature of this study is that individual AI risk prediction charts are provided, making clear which parameters contributed to the classification. A further step has been to use ML for major adverse cardiac event (MACE) prediction. On a very large proportion of REFINE patients, the Authors explored various ML methodologies and determined the most effective set of imaging and clinical variables to achieve a MACE prediction that was superior to those of stress TPD and of traditional multivariable models [47]. Although the best prediction was reached using the full variable panel, reduced and more practical models obtained clinically valuable results. Similarly, Betancur and coworkers explored the predictive value of ML in a cohort of 2619 patients and demonstrated that the ML combination of imaging and clinical features was superior to ML imaging alone and to the other traditional methods [48]. These results were confirmed in a much larger patient cohort of over 20,000 patients by Singh and coworkers, who developed an explainable DL model for predicting MACE, which obtained an AUC of 0.73 compared with the 0.70 of a logistic regression model and 0.65 of stress TPD [49]. The explainability of the patient classification is another major merit of this article. Pieszko and coworkers performed a complex predictive study focusing separately on three different adverse outcome categories (all cause death, acute coronary syndrome, and revascularization), trying as well to derive from the model a time-to-event outcome prediction. Moreover, an individual prediction was obtained in the form of a waterfall plot [50]. Using ML for clustering analysis, Williams et al. were able to identify three clusters with significantly different outcomes from a REFINE subpopulation, including more than 9000 patients with known CAD [51]. A most recent and intriguing paper about the potential of AI for prognostication has demonstrated that it is possible to identify patient clusters at higher risk for cardiovascular events even among MPI studies classified as normal [52].

**Table 1:** Summary of the most important articles using AI in nuclear cardiology. Ref.: reference number; * = clinical data evaluated together with MPI.

Ref.	Main Author/Year	AI Method	Input Data Type	Dataset Size	Accuracy	AUC	REFINE	Study Purpose	Reference Standard
[26]	Arsanjani/2013	LogitBoost	SPECT MPI *	1181	87.3%	0.94	no	Diagnostic	TPD and expert reading
[27]	Berkaya/2020	DL-based and knowledge- based models.	SPECT MPI	192	94% (DL-based); 93% (K-based)	n.r.	no	Diagnostic	Expert reading
[28]	Apostolopoulos/2021	CNN + RF	SPECT MPI *	566	78,44%	0.7926	no	Diagnostic	ICA + expert reading
[29]	de Souza Filho/2021	ML ensemble (AB, GB, RF, XGB)	SPECT MPI	1007	93.8% RF	0.853 RF	no	Diagnostic	n.r.
[30]	Miller/2022	Grad-CAM	SPECT MPI *	828 training, 511 test	n.r.	0.93	yes	Diagnostic	uTPD and sTPD
[31]	Rios/2022	XGBoost, RF	SPECT MPI *	20,179	n.r.	0.799	yes	Prognostic	Stress TPD and expert reading
[35]	Miller/2025	TPD-DL and SSS-DL	SPECT MPI	555	n.r.	0.837	yes	Diagnostic	Stress TPD and SSS
[36]	Miller/2022	CAD-DL	SPECT MPI *	240 patients	n.r.	0.779	no	Diagnostic	Expert reading and stress TPD
[37]	Zhang/2024	DL	SPECT MPI	1038	88.7%	0.82	no	Diagnostic	Expert reading
[40]	Betancur/2018	CNN	SPECT MPI	1638	n.r.	0.80	yes	Diagnostic	Stress TPD
[41]	Betancur/2019	CNN	SPECT MPI	1160	n.r.	0.81	yes	Diagnostic	cTPD
[42]	Liu/2021	CNN	SPECT MPI	37,243	82.7%	0.872	no	Diagnostic	Quantitative perfusion defect size
[43]	Miller/2022	XGBoost	SPECT MPI *	20,418 train, 9019 test	n.r.	0.762	yes	Prognostic	Clinical CAD consortium
[44]	Otaki/2022	CAD-DL	SPECT MPI *	3578	n.r.	0.83	yes	Diagnostic	Stress TPD and expert reading
[45]	Hu/2020	LogitBoost (ensemble ML)	SPECT MPI *	1980	n.r.	0.81	yes	Prognostic	Stress TPD and ischemic TPD
[46]	Feher/2024	XGBoost	SPECT MPI *	4766 train, 2912 test	n.r.	0.87	yes	Prognostic	Stress TPD and stress LVEF
[47]	Rios/2022	Multiple ML	SPECT MPI *	20,414 train, 2984 test	n.r.	0.755	yes	Prognostic	Stress TPD and expert reading
[48]	Betancur/2018	Boosted ensemble	SPECT MPI *	2619	n.r.	0.81	no	Prognostic	Stress TPD and expert reading
[49]	Singh/2023	HARD MACE-DL	SPECT MPI *	20,418 train, 9019 test	n.r.	0.73	yes	Prognostic	Logistic regression model and stress TPD
[50]	Pieszko/2023	Time-to-event DL	SPECT MPI *s	20,418 train, 13,988 test	n.r.	0.76 for ACS, 0.78 all-cause death	yes	Prognostic	n.r.
[53]	Juarez-Orozco/2020	LogitBoost (ensemble ML)	13N-PET *	1234	n.r.	0.72 (ischemia), 0.71 (MACE)	no	Diagnostic and Prognostic	Logistic regression and SCORE risk model
[54]	Berman/2024	Multiple ML	82Rb-PET	3245	n.r.	0.95	no	Diagnostic	Standard LR
[55]	Juarez-Orozco/2020	ResNet50	13N-PET	1185	n.r.	0.90	no	Prognostic	Integrated model
[56]	Singh/2022	Grad-CAM	82Rb-PET	4735	n.r.	0.82	no	Prognostic	Ischemia, MFR, logistic regression
[57]	Kwiecinski/2022	XGBoost	18F-NaF PET *	293	n.r.	0.85	no	Prognostic	Quantitative plaque analysis

Other studies have explored the potential of AI approaches applied to myocardial positron emission tomography (PET). Juarez-Orozco and coworkers used quantitative PET data as reference to classify the patients as ischemic (regional myocardial perfusion reserve—MPR < 2.0) or as at high risk (global MPR < 2.0). Then they explored the capability of ML to predict these two PET classifications based on demographic, clinical, and functional variables, achieving an acceptable AUC [53]. Berman et al. demonstrated good results of a relatively simple ML model applied to ^82^Rb quantitative myocardial perfusion PET for detection and localization of perfusion abnormalities. A more complex approach did not significantly improve the results [54]. An interesting further step has been to use DL for MACE prognostication. The predictive power of the DL approach was compared with that of different traditional models (clinical, functional, absolute perfusion quantification, and of their integration). DL achieved the largest AUC (0.90), whilst the integrated approach reached 0.85 [55]. Singh and coworkers applied DL to 4735 patients submitted to ^82^Rb PET, obtaining an AUC of 0.82 compared with myocardial flow reserve (AUC = 0.70) and a comprehensive logistic regression model (AUC = 0.75) [56].

### 2.5. ML and DL: AI Applications Beyond MPI

The usefulness of AI was also explored in the setting of other cardiological PET investigations. For instance, Kwiecinski and coworkers used 18F-NaF PET coupled with CT angiography and demonstrated that a ML model integrating clinical, CT plaque, and 18F-NaF PET variables reached an AUC = 0.85 for predicting subsequent myocardial infarction [57].

Another field of potential applications of AI is the detection of cardiac uptake of bone tracers in patients with Transthyretin Amyloidosis (ATTR). In a first experience, Delbarre and coworkers used a convolutional neural network on a training set of more than 3000 images and then on a validation set of 1633 images, with very good results (AUC = 0.999) [58]. Similarly, quite effective results for detecting ATTR in bone scans performed for the usual indications were obtained by Halme and coworkers, who evaluated 1334 patients and demonstrated an AUC of >0.85 for detecting ATTR uptake [59]. In a very complex study, Salimi and coworkers examined a ML model for ATTR detection and scoring, which performed quite well in the external datasets of patients suspicious of ATTR. On the other hand, in a further large dataset of patients screened for other indications, the results were quite disappointing, with just ten suspicious cases detected and four of them reclassified as false positives by an expert review [60]. Better results have been reported by Spielvogel and coworkers, who examined a very large dataset (16,241 patients with 19,401 images). Their results were excellent for the diagnosis of cardiac ATTR, with a very high AUC. Even for the screening task, the results were satisfactory, with a very limited rate of false positive and false negative classifications. Moreover, a positive result achieved by the AI model showed a significant predictive value for the subsequent mortality and for heart failure hospitalizations [61]. DL has been effectively used to perform an automated quantification of 99mTc-PYP in patients with suspected ATTR submitted to SPECT [62].

Finally, it is worth mentioning the study by Togo and coworkers for applying a convolutional neural network in the detection of cardiac sarcoidosis using [^18^F]-FDG, obtaining a gain in comparison to the standard SUV-based approaches [63].

## 3. Final Remarks

### 3.1. The Current Status

The great interest in AI applications to the field of cardiology is confirmed by the steady increase in articles reporting AI-based models for diagnosis and prognosis, and nuclear cardiology is fully involved in this process. The multiple advantages of AI are well-known and include the capability of extracting hidden information both from images and patient characteristics, to handle big data, to integrate clinical and imaging features, and to offer an objective basis for physician’s interpretation.

On the other hand, the current hype about AI should not let us neglect its current limitations and the problems related to its use. So far, the gains in terms of diagnostic accuracy remain relatively small, despite the use of different AI-based algorithms. The causes are mainly methodological problems, such as inadequate patient populations, both owing to small cohorts or unbalanced distribution, inherent difficulties for selecting a reference standard, and last, but not least, the already good performance of the established methods. The use of AI-based models appears more effective for prognostication, because there are no problems with reference standard, and the integration of imaging with multiple other clinical data is a major strength of AI. On the other hand, before being confidently used, AI risk stratification should be prospectively evaluated.

**Figure 3:**
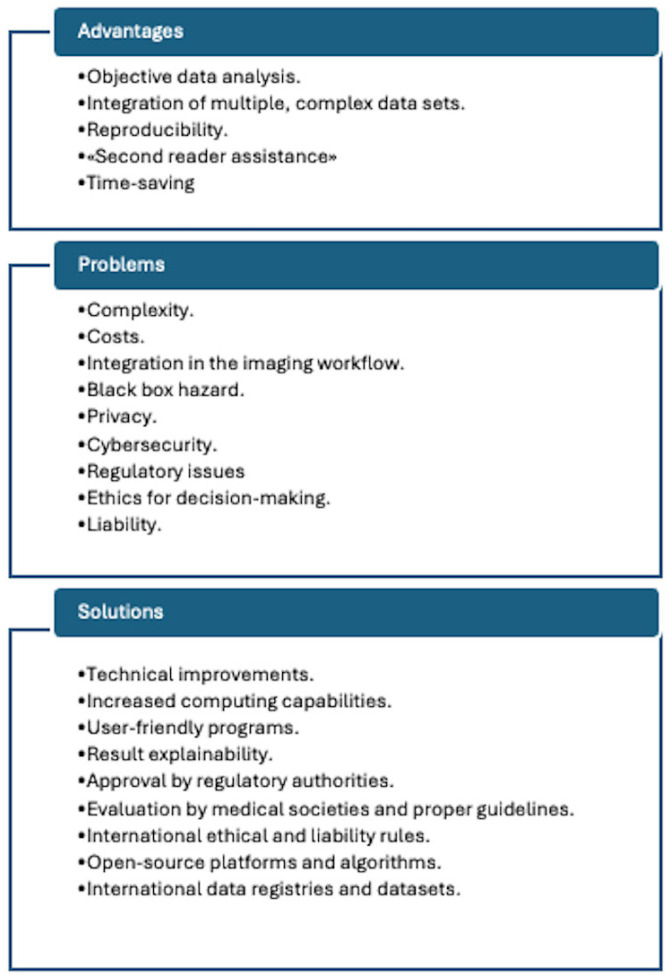
Summary of present problems and future perspectives for AI in nuclear cardiology, as suggested by reference [22].

As a matter of fact, presently none of the many proposed AI algorithms have become part of the current clinical routine. To reach this goal several steps are still needed. Conversely, the introduction of AI-based models in medical practice poses a series of other important problems, besides that of operative feasibility. Figure 3 depicts various relevant points, together with some proposed solutions.

### 3.2. Future Perspectives

The first step for a wide use of AI in nuclear cardiology would be the implementation of the selected algorithms in one of the currently employed programs. The simplest scenario could be to have AI-based scores, such as SSS or TPD, as an alternative to the standard calculation. Another quite feasible approach could be the construction and diffusion of AI-based risk stratification software. Both instances, however, would probably require the involvement of commercial actors, such as software houses or device vendors. This step outside the research world poses a series of potential problems in terms of ethics and regulations. Among them are the protection of patient personal data, which would become the prerequisite for the AI model, the cybersecurity of the program, and the costs for development and implementation. Moreover, regulatory problems would arise, with the need of approval of the program by a competent authority, a quite complex issue given the peculiarity of AI algorithms as compared to drugs or medical devices. In other fields of medical imaging, namely radiology, various statements have been already published about these issues [64,65].

The ethical concerns related to the use of AI for clinical decision-making remain unsolved and will likely soon be the most delicate ones. They involve the behaviour of the single physician as well as more general liability questions. For the clinicians, the explainability of the AI-derived patient classification is the most important condition for a reliable use. Ideally, what is needed is not just some kind of visual representation of the model results but a clear interpretation of their meaning, making it therefore possible to transfer it to the patient as well. Patient involvement through his/her comprehension of the technique is necessary to make acceptable the ensuing therapeutic choices.

The inclusion of AI models in the clinical decision-making will require attentive proof by medical societies. Moreover, the liability for the consequences of AI-based options remains to be clearly defined. The issue could become particularly difficult if AI-based classification is adopted without being previously interpreted by a physician. In this case it must be settled how the responsibility should be divided between AI (and thus its developers) and the clinician who uses it.

All the above-mentioned concerns could become even more complex with the evolution of AI and the expansion of its applications, as for instance text-generating chatbots.

The highest awareness on these matters by the medical community is a prerequisite for an effective and safe use of AI in nuclear cardiology.

## Abbreviations

The following abbreviations are used in this manuscript:

AI	Artificial Intelligence
ATTR	Transthyretin Amyloidosis
AUC	Area Under Curve
CAD	Coronary Artery Disease
CNN	Convolutional Neural Network
CT	Computed Tomography
CZT	Cadmium-Zinc-Telluride
DL	Deep Learning
MACE	Major Adverse Cardiac Event
ML	Machine Learning
MPI	Myocardial Perfusion Imaging
MRI	Magnetic Resonance Imaging
PET	Positron Emission Tomography
SDS	Summed Difference Score
SPECT	Single-Photon Emission Computed Tomography
SSS	Summed Stress Score
TPD	Total Perfusion Defect
